# Risk Factors and Trends Associated With Mortality Among Adults With Hip Fracture in Singapore

**DOI:** 10.1001/jamanetworkopen.2019.19706

**Published:** 2020-02-14

**Authors:** Eu-Leong Yong, Ganga Ganesan, Michael S. Kramer, Tet Sen Howe, Joyce S. B. Koh, Win Pa Thu, Susan Logan, Jane A. Cauley, Kelvin B. Tan

**Affiliations:** 1 Department of Obstetrics and Gynecology, National University Hospital, National University of Singapore, Singapore; 2 Division of Policy, Research and Evaluation, Ministry of Health, Singapore; 3 Department of Epidemiology, Biostatistics and Occupational Health, McGill University Faculty of Medicine, Montreal, Quebec, Canada; 4 Department of Pediatrics, McGill University Faculty of Medicine, Montreal, Quebec, Canada; 5 Department of Orthopedic Surgery, Singapore General Hospital, Singapore; 6 Department of Epidemiology, University of Pittsburgh Graduate School of Public Health, Pittsburgh, Pennsylvania; 7 Saw Swee Hock School of Public Health, National University of Singapore, Singapore

## Abstract

**Importance:**

Examining trends in mortality following hip fracture and its associated factors is important for population health surveillance and for developing preventive interventions.

**Objective:**

To examine temporal trends in, and risk factors associated with, mortality following hip fracture over 18 years in Singapore.

**Design, Setting, and Participants:**

This retrospective, population-based cohort study included men and women aged 50 years and older admitted to Singapore hospitals for first hip fracture identified and followed up from 2000 to 2017. Demographic information, fracture type, and Charlson Comorbidity Index (CCI) score were retrieved from nationwide claims data, and mortality data were from the National Death Registry. Data were analyzed from August 2018 to December 2019.

**Main Outcomes and Measures:**

Adjusted hazard ratios (aHRs) and their 95% confidence intervals were estimated using Cox proportional hazards regression. Kaplan-Meier life table methods were used to calculate survival following the hip fracture on a cohort basis. The crude survival over time since fracture was compared by sex, age group, ethnicity, CCI, and fracture type. Standardized mortality ratios (SMRs) were calculated using all-cause mortality obtained from Singapore population life tables.

**Results:**

Among 36 082 first inpatient admissions for hip fractures (mean [SD] patient age, 78.2 [10.1] years; 24 902 [69.0%] female; 30 348 [84.1%] Chinese, 2863 [7.9%] Malay, 1778 [4.9%] Indian, and 1093 [3.0%] other ethnicity), elevated rates of mortality were observed for male sex (aHR, 1.46; 95% CI, 1.41-1.52), Malay ethnicity (aHR, 1.23; 95% CI, 1.15-1.30 vs Chinese ethnicity), older age (aHR, 5.20; 95% CI, 4.27-6.34 for age ≥85 years vs 50-54 years), high CCI score (aHR, 3.62; 95% CI, 3.42-3.84 for CCI ≥6 vs CCI of 0), trochanteric fractures (aHR, 1.11; 95% CI, 1.06-1.16 vs cervical fractures), and earlier cohorts (aHR, 0.59; 95% CI, 0.56-0.62 for 2012-2017 vs 2000-2005). Absolute mortality decreased significantly over time: by 21% in 2006 to 2011 and by 40% in 2012 to 2017, compared with 2000 to 2005. On long-term follow-up, differences in survival associated with sex and ethnicity tended to diminish, whereas differences associated with older age, higher CCI score, and trochanteric fractures increased. In the first year after fracture, reductions in SMR were observed comparing the periods 2013 to 2016 with 2003 to 2007 in women (SMR, 2.05; 95% CI, 1.91-2.20 vs SMR, 2.54; 95% CI, 2.39-2.70, respectively) but not among men (SMR, 3.28; 95% CI, 3.04-3.54 vs SMR, 3.42; 95% CI, 3.18-3.68, respectively).

**Conclusions and Relevance:**

Malay ethnicity, older age, male sex, prefracture comorbidity, and trochanteric fractures were independently associated with increased risk of death, identifying population groups that could be targeted for intervention strategies. The improvement in relative mortality for women but not men suggests the need to develop interventions that improve mortality outcomes for men.

## Key Points

**Question:**

What factors are associated with long-term mortality after hip fracture in a multiethnic Asian population?

**Findings:**

In this population-based cohort study of 36 082 patients in Singapore who experienced hip fractures from 2000 to 2017, Malay ethnicity, older age, male sex, prefracture comorbidity, and trochanteric fractures were independently associated with increased risk of death. Absolute mortality decreased significantly over time, whereas reduction in standardized mortality ratio was observed among women in the first 4 years after fracture, but not among men.

**Meaning:**

These findings suggest that interventions to improve outcomes after hip fracture need to focus on patients who are male, are of Malay ethnicity, have higher prefracture comorbidity, and have trochanteric fractures.

## Teaser

This population-based cohort study examines risk factors and trends associated with mortality following hip fracture among older adults in Singapore.

## Introduction

Hip fracture is a devastating event associated with a high risk of death. Approximately 5% of all-cause mortality is attributable to hip fracture.^1^ While fracture rates have decreased in most advanced nations, absolute numbers continue to increase because of aging populations.^2^ Studies^3,4^ have documented increased risk for death in the first few years after injury. However, data on prognosis for the longer term (5-10 years) are relatively scarce: a systematic review^3^ located only 5 long-term studies, all in Caucasian populations. Cohort studies^5,6,7,8^ suggest that the excess mortality after hip fractures may be attributable to increased frailty, poor nutritional status, lower level of physical activity, and worsening comorbid conditions such as chronic liver, kidney, or cardiovascular diseases and pneumonia and dementia. Examining long-term trends and understanding the causes of mortality following hip fracture are important for population health service planning and for developing preventive interventions.^9,10,11^

Although the age-adjusted rate of hip fracture has decreased 1.4% annually in Singapore, an increase of 72 additional fractures per 100 000 per year for the past 18 years reflects the aging of its population.^12^ The only previous Singapore study of hip fracture mortality was restricted to Chinese patients and limited to 5 years of follow-up.^13^ Individuals of Chinese, Indian, and Malay ethnicities make up more than 40% of the global population.^14^ Singapore is an island nation whose population largely comprises these 3 ethnic groups. Knowledge of risk factors and trends underpinning long-term mortality after hip fracture in Singapore should therefore apply not only to Singapore, but also to Chinese, Malay, and Indian populations in other urban areas of East, Southeast, and South Asia.

In this study, we examined mortality for up to 18 years following hip fracture and its association with ethnic, demographic, and comorbidity risk factors in the Singapore population. We also assessed trends in mortality after fracture compared with mortality in the general population.

## Methods

The study population comprised patients aged 50 years or older admitted to Singapore hospitals for first hip fracture during the 18-year period from 2000 to 2017, as described in a previous study.^12^ The administrative data sets from the Ministry of Health include all records of all inpatient episodes that are submitted for Medisave and MediShield claim purposes. Claims are based on a unique National Registration Identification Number issued at birth (or, for persons born outside of Singapore, at the time of obtaining residency status) and also include the ethnicity of patients. All analyses were performed using anonymized data deidentified by the Ministry of Health, Singapore. Waiver of informed consent requirements and approval of the study were granted by the National Healthcare Group, Domain Specific Review Board. Reporting of this study follows the Strengthening the Reporting of Observational Studies in Epidemiology (STROBE) reporting guideline. Data were analyzed from August 2018 to December 2019.

Hip fractures were identified using inpatient diagnostic codes from the *International Statistical Classification of Diseases and Related Health Problems, Tenth Revision*, Australian version (ICD-10-AM): S7200, S7201-S7211, and S722-S723 for the period 2012 to 2015; and from the *International Classification of Diseases, Ninth Revision, Clinical Modification*, Australian version (ICD-9-CM): 820, 820.0, 820.2, and 820.8 for the period 2000 to 2011. To ensure that only first episodes of hip fracture were captured, persons with a hip fracture during the preceding 5 years (1995-1999) were excluded. Demographic information (age, sex, and ethnicity) was obtained from claims data. The Charlson Comorbidity Index (CCI) score for each patient was calculated based on preexisting comorbidities identified from diagnostic codes obtained from the Ministry of Health’s nationwide administrative databases on inpatient admissions, day surgery and emergency department episodes, primary health care clinic visits (polyclinics), and Community Health Assist Scheme general practitioner clinic visits databases. Mortality data from both patients with hip fracture and the general population were obtained from the National Death Registry. Housing categories (public or private) were inferred from residential postal codes.

### Statistical Analysis

We used Kaplan-Meier life table methods to calculate survival following hip fracture on a cohort basis for each 3-month period in the first year following hip fracture and yearly thereafter. We had a maximum of 18 years of follow-up for fractures occurring in 2000, and a minimum of 1 month follow-up for fractures occurring in 2017. We then produced graphs to compare the crude survival over time since fracture by sex, age group (in 5-year categories beginning at 50-54 years and terminating at ≥85 years), ethnicity (Chinese, Malay, Indian, or other), CCI score (0, 1-3, 4-5, or ≥6), and fracture type (trochanteric, cervical, or other). Factors independently associated with mortality were analyzed by estimating adjusted hazard ratios (aHRs) and their 95% confidence intervals using Cox proportional hazards regression.

Expected mortality was based on the annual mean age- and sex-specific probability of all-cause mortality obtained from Singapore Population Life Tables 2003 to 2016.^15^ We therefore limited relative survival analyses (standardized mortality ratios [SMRs]) to the years 2003 to 2016, calculated as the observed mortality for each sex and age group divided by the expected mortality for that same group in each year. Sampling variability (precision) around the estimated SMRs are denoted by their 95% confidence intervals.

## Results

We ascertained 36 082 first inpatient admissions (mean [SD] patient age, 78.2 [10.1] years; 24 902 [69.0%] female) for hip fractures in Singapore in the 18-year period from 2000 to 2017. A flow diagram of cohort patients has been presented in our previous publication.^12^ As shown in Table 1, 10.7% of the fractures occurred in persons from 50 to 64 years of age, while 29.2% occurred in those aged 85 years or older. In all, 30 348 patients (84.1%) were of Chinese ethnic origin, followed by Malay (2863 [7.9%]), Indian (1778 [4.9%]), and other (1093 [3.0%]) ethnicities. Men were 3.6 years younger (difference in means) than women at the time of fracture. The majority of patients (87.1%) resided in state-subsidized public housing. Trochanteric fractures made up 46.7% of the fractures, with cervical fractures accounting for 30.7%. In all, 25.5% of patients had no comorbidity (CCI score = 0), while 33.3% had a CCI score of 4 or higher.

**Table 1.** Patient Characteristics

Characteristic	No. (%)		
	Total (N = 36 082)	Men (n = 11 180)	Women (n = 24 902)
Age, y			
Mean (SD)	78.2 (10.1)	75.8 (10.7)	79.4 (9.7)
50-54	679 (1.9)	417 (3.7)	262 (1.1)
55-59	1149 (3.2)	580 (5.2)	569 (2.3)
60-64	2035 (5.6)	832 (7.4)	1203 (4.8)
65-69	3145 (8.7)	1223 (10.9)	1922 (7.7)
70-74	4772 (13.2)	1637 (14.6)	3135 (12.6)
75-79	6554 (18.2)	1992 (17.8)	4562 (18.3)
80-84	7224 (20.0)	2016 (18.0)	5208 (20.9)
≥85	10 524 (29.2)	2483 (22.2)	8041 (32.3)
Charlson Comorbidity Index score			
0	9208 (25.5)	2478 (22.2)	6730 (27.0)
1-3	14 880 (41.2)	4433 (39.7)	10 447 (42.0)
4-5	5399 (15.0)	1789 (16.0)	3610 (14.5)
≥6	6595 (18.3)	2480 (22.2)	4115 (16.5)
Housing type^a^			
Public, 1-2 rooms	3033 (9.0)	1078 (10.5)	1955 (8.4)
Public, 3 rooms	9660 (28.7)	3090 (30.1)	6570 (28.1)
Public, 4-5 rooms	16 628 (49.4)	4932 (48.1)	11 696 (50.0)
Private	4336 (12.9)	1150 (11.2)	3186 (13.6)
Ethnicity			
Chinese	30 348 (84.1)	9084 (81.3)	21 264 (85.4)
Malay	2863 (7.9)	950 (8.5)	1913 (7.7)
Indian	1778 (4.9)	744 (6.7)	1034 (4.2)
Other	1093 (3.0)	402 (3.6)	691 (2.8)
Fracture type			
Cervical	11 094 (30.7)	2973 (26.6)	8121 (32.6)
Trochanteric	16 841 (46.7)	6197 (55.4)	10 644 (42.7)
Other or unspecified	8147 (22.6)	2010 (18.0)	6137 (24.6)

^a^ Data were missing for 2425 patients (6.7%).

**Figure 1.**
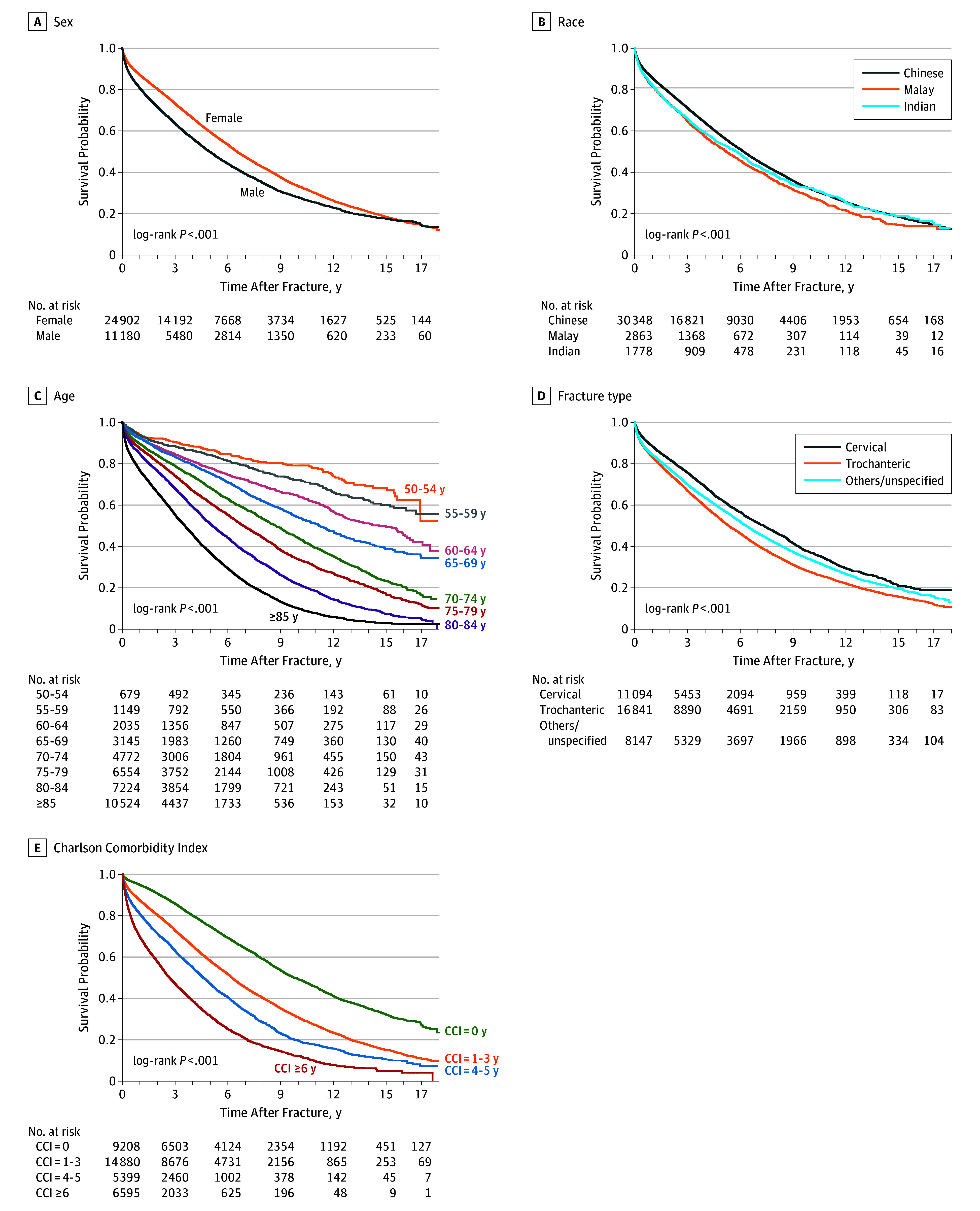
Survival in 36 082 Patients up to 18 Years Following Hip Fracture According to Sex, Ethnicity, Age, Fracture Type, and Charlson Comorbidity Index (CCI) Score

Figure 1 compares the yearly survival rates following hip fracture by sex, ethnicity, age, CCI group, and fracture type. Survival rates were lower among men, Malay individuals, older age groups, groups with high CCI scores, and those with trochanteric fractures. Differences in survival associated with sex and ethnicity tended to diminish with long-term follow-up. In contrast, differences in survival associated with older age, high CCI score, and trochanteric fractures increased over time. Table 2 summarizes the results of the Cox regression analysis. Factors independently associated with significantly elevated hazard ratios for mortality were male sex (aHR, 1.46; 95% CI, 1.41-1.52), Malay ethnicity (aHR, 1.23; 95% CI, 1.15-1.30 vs Chinese ethnicity), older age (aHR, 5.20; 95% CI, 4.27-6.34 for age ≥85 years vs 50-54 years), high CCI score (aHR, 3.62; 95% CI, 3.42-3.84 for CCI ≥6 vs CCI of 0), trochanteric fractures (aHR, 1.11; 95% CI, 1.06-1.16 vs cervical fractures), and earlier cohorts (aHR, 0.59; 95% CI, 0.56-0.62 for 2012-2017 vs 2000-2005). High socioeconomic status, as represented by private housing, was significantly associated with reduced mortality in these adjusted analyses. Mortality decreased by 21% in 2006 to 2011 and by 40% in 2012 to 2017 compared with 2000 to 2005.

**Table 2.** Hazard Ratios for Mortality After Hip Fracture Based on Multivariable Cox Regression

Characteristic	Hazard Ratio (95% CI)
Male	1.46 (1.41-1.52)
Ethnicity	
Chinese	1 [Reference]
Malay	1.23 (1.15-1.30)
Indian	1.03 (0.96-1.11)
Other	1.09 (0.99-1.20)
Age group, y	
50-54	1 [Reference]
55-59	0.96 (0.76-1.21)
60-64	1.28 (1.04-1.59)
65-69	1.47 (1.20-1.80)
70-74	1.94 (1.59-2.37)
75-79	2.51 (2.06-3.06)
80-84	3.40 (2.79-4.15)
≥85	5.20 (4.27-6.34)
Charlson Comorbidity Index category	
0	1 [Reference]
1-3	1.45 (1.38-1.51)
4-5	2.20 (2.09-2.32)
≥6	3.62 (3.42-3.84)
Housing category	
Public, 1-2 rooms	1 [Reference]
Public, 3 rooms	1.02 (0.95-1.09)
Public, 4-5 rooms	1.00 (0.94-1.06)
Private	0.92 (0.87-0.98)
Cohort	
2000-2005	1 [Reference]
2006-2011	0.76 (0.73-0.79)
2012-2017	0.59 (0.56-0.62)
Fracture type	
Cervical	1 [Reference]
Trochanteric	1.11 (1.06-1.16)
Other or unspecified	1.07 (1.02-1.13)

**Figure 2.**
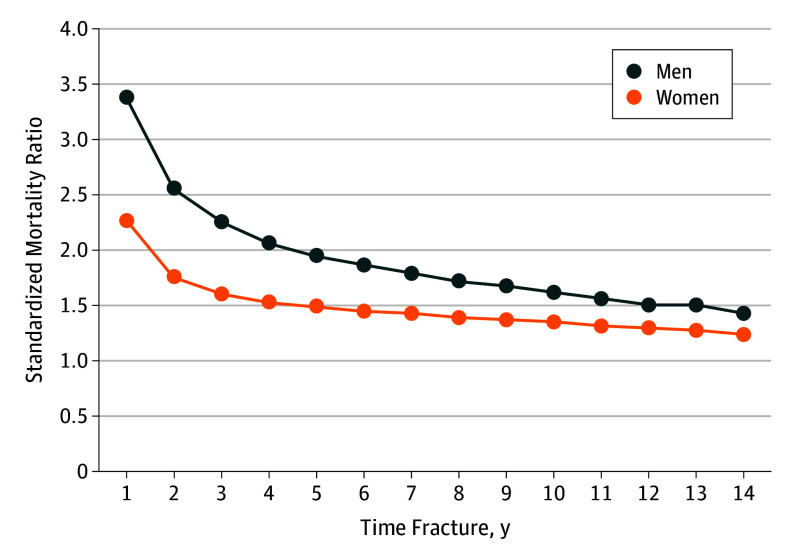
Relative Mortality in Men and Women After Hip Fracture

As shown in Figure 2 and the eTable in the Supplement, relative mortality was consistently higher in men for years 1, 2, 5, 10, and 14 following fracture (SMR 3.4, 2.6, 1.9, 1.6, and 1.4, respectively) than in women (SMR 2.3, 1.7, 1.5, 1.3, and 1.2, respectively). In the first year after fracture, reductions in SMR were observed comparing the periods 2013 to 2016 with 2003 to 2007 among women (SMR, 2.05; 95% CI, 1.91-2.20 vs SMR, 2.54; 95% CI, 2.39-2.70, respectively), but not among men (SMR, 3.28; 95% CI, 3.04-3.54 vs SMR, 3.42; 95% CI, 3.18-3.68, respectively) (Table 3). In the first year after fracture, the youngest age group (50-64 years) had the highest SMR (12.4 [95% CI, 10.2-15.1] in women and 10.6 [95% CI, 8.9-12.5] in men) compared with those aged 65 years and older (2.1 [95% CI, 2.1-2.2] in women and 3.1 [95% CI, 3.0-3.3] in men) (eFigure in the Supplement). Younger patients also experienced the sharpest decrease over the first 3 to 5 years after fracture. When we examined trends across 3 different cohorts defined by calendar time (2003-2007, 2008-2012, and 2013-2016), a reduction in SMR was observed across the 3 cohorts among women in the first 4 years after fracture, but not among men (Table 3).

**Table 3.** Trends in Standardized Mortality Ratio Across 3 Periods in Men and Women During the First 4 Years After Hip Fracture

Period	Year After Fracture	Standardized Mortality Ratio (95% CI)^a^	
		Men	Women
2003-2007	1	3.42 (3.18-3.68)	2.54 (2.39-2.70)
	2	2.53 (2.39-2.68)	1.84 (1.75-1.93)
	3	2.21 (2.11-2.32)	1.64 (1.58-1.71)
	4	2.03 (1.95-2.11)	1.55 (1.50-1.61)
2008-2012	1	3.39 (3.16-3.65)	2.19 (2.06-2.34)
	2	2.56 (2.42-2.71)	1.73 (1.65-1.81)
	3	2.22 (2.11-2.33)	1.59 (1.53-1.66)
	4	2.08 (1.99-2.16)	1.53 (1.48-1.58)
2013-2016	1	3.28 (3.04-3.54)	2.05 (1.91-2.20)
	2	2.59 (2.44-2.75)	1.67 (1.58-1.76)
	3	2.32 (2.20-2.45)	1.57 (1.50-1.65)
	4	2.09 (1.98-2.20)	1.48 (1.42-1.55)

^a^ Standardized mortality ratios refer to the years listed; they are not cumulative.

## Discussion

Despite the increasing burden of hip fractures associated with rapidly aging populations, few studies of hip fracture mortality from Asia have been published. The most recent meta-analysis^3^ of mortality following hip-fracture did not include any studies based on Asian populations. Published Asian studies^16,17^ have follow-up periods only up to 2 years, with the exception of a single study^18^ from Taiwan of patients with hip fractures who underwent surgery, which followed up patients for 10 years, and 1 study^13^ from Singapore restricted to Chinese patients and limited to 5 years of follow-up. To our knowledge, our large population-based study is the first from Singapore and allows us to report risk of mortality beyond 10 years after hip fracture. Male sex, Malay ethnicity, older age, high CCI score, and trochanteric fractures were all independently associated with elevated risk for mortality.

Our results are consistent with the widely recognized observation that men have higher mortality after hip fracture than women.^3,19^ Higher mortality in men compared with women cannot be explained by differences in quality of in-hospital care^20^ and remains even after controlling for age, fracture site, number of medications, and chronic comorbidities.^19,21^ In our analysis, men had a 46% higher risk of death than women, even after adjusting for comorbid conditions.

Increasing morbidity, as measured by higher CCI scores, was independently associated with mortality in our study. Survival disparities associated with CCI scores remained even 18 years after the index fracture. Of course, comorbid conditions increase mortality in the absence of fracture. Excess mortality associated with hip fracture was attenuated when adjusted for CCI score in an Estonian cohort.^22^ On the other hand, prefracture comorbidity remained independently associated with mortality over 6 years in a large Norwegian hip fracture cohort^23^ matched by comorbidity with control participants without hip fracture. One challenge for future work in Singapore is to compare hip fracture mortality with mortality in a nonfracture cohort matched by CCI score.

Compared with cervical fractures, trochanteric fractures are associated with advanced age and with higher CCI score,^24^ implying a more fragile group of patients. Hips fracturing at the trochanteric region have been documented to have reduced cortical thickness,^25^ bone mineral density, and bone mechanical strength.^26^ These fractures can vary in morphology, ranging from simple fractures to highly comminuted, unstable configurations. As these fractures are usually treated with fixation, patients may not be able to bear weight immediately. Longer time from operation to mobilization has been associated with increased rates of complications and mortality after hip fractures.^27^ Thus, the association of trochanteric fractures with higher mortality is likely due to its occurrence in older, sicker patients with poorer bone quality.^28^

Absolute hip fracture mortality has decreased by 20% to 40% over the past 15 years. This improvement, however, may largely be driven by decreases in mortality in the general population. Mortality decreased steadily during the period of 2000 to 2017, with life expectancy improving by 5.2 years for women and 4.7 years for men.^15^ When we examined time trends in hip fracture SMR, we observed stable relative mortality in men but a decrease in women. These results differ from those reported in a study^1^ from Norway, where relative mortality increased over time for women but not for men. The decrease we observed in female SMR occurred mostly during the first year after fracture, with smaller decreases observed in subsequent years. The reasons for geographic and sex differences in trend require further study.

Strengths of our study include its prolonged period of follow-up and its use of national administrative databases, which ensures complete capture and follow-up of all hip fractures. We also compare outcomes of patients with hip fracture against population mortality rates from published life tables. Other studies may introduce selection bias by using control groups that may not be representative of the general population. Another strength is our use of several longitudinal databases to capture comorbidities over time, not just at the time of hip fracture. Studies based on selected hip fracture cohorts (ie, not population based) may suffer from selection bias. For instance, a Taiwan study^18^ reported only on patients who underwent surgery, and its results may therefore reflect changes in clinical decision-making and surgery rates over time. Nevertheless, the SMRs of patients with hip fractures in Singapore are lower than those reported in Taiwan,^18^ even though the Taiwan study was restricted to surgical cases and despite the higher comorbidity of the patients with hip fracture in our study. Our estimated SMRs are also lower than those reported in the meta-analysis by Haentjens et al.^3^ Whether this is attributable to better care of patients with hip fracture or differences in underlying patient characteristics requires further study.

### Limitations

This study has some limitations. As with many previous studies on relative mortality, the key limitation of our study is that we have only been able to compare the mortality of patients with hip fracture with that of a general population of similar age groups and sex. The elevated mortality over the long term may simply be due to the increased mortality associated with the comorbidities of patients with hip fracture rather than the hip fracture itself.^19,21^

## Conclusions

Life spans are increasing globally, thereby increasing the cumulative (lifetime) risk of hip fracture and its fatal consequences. Despite improving absolute mortality outcomes, the elevated mortality risk compared with the general population (SMR) implies that efforts to prevent hip fractures, such as fall prevention and prophylactic medication among elderly individuals with osteoporosis, may be cost-effective by reducing the mortality burden of hip fracture. The mortality risk factors we identified—older age, male sex, prefracture comorbidity, and Malay ethnicity—help to identify a target population for cost-effective prevention strategies. For instance, while our previous work^12^ identified a lower prevalence of hip fractures in Malay individuals compared with other ethnic groups, their higher mortality may justify preventive strategies in that ethnic group. To our knowledge, differences in risk of death after hip fracture among Chinese, Malay, and Indian adults have not been previously reported. Differences by comorbidity are of interest in themselves, have not been widely reported in other populations, and also show that other (better-known) differences persist after controlling for the confounding effect of comorbidity. Our study provides encouraging results that absolute mortality after hip fracture has decreased over time, as has short-term relative mortality in women. Future research should attempt to develop and test interventions to reduce the fatality rate of hip fractures, especially in men.

## Notes

Supplement. **eTable.** Absolute and Relative Mortality (SMR) Over Each Time Interval After Hip Fracture **eFigure.** Age and Relative Mortality (Standardized Mortality Ratio) After Hip Fracture in A) Men and B) Women.
