# Supplementary material for: Risk Factors and Trends Associated With Mortality Among Adults With Hip Fracture in Singapore
Source: JAMA Netw Open. 2020 Feb 14;3(2):e1919706. doi: 10.1001/jamanetworkopen.2019.19706 (PMC12124694; doi:10.1001/jamanetworkopen.2019.19706)
Supplement: Supplement. — eTable. Absolute and Relative Mortality (SMR) Over Each Time Interval After Hip Fracture eFigure. Age and Relative Mortality (Standardized Mortality Ratio) After Hip Fracture in A) Men and B) Women. [file jamanetwopen-e1919706-s001.pdf]

Supplementary Online Content

Yong E-L, Ganesan G, Kramer MS, et al. Risk factors and trends associated with mortality among adults with hip fracture in Singapore. *JAMA Netw Open*. 2020;3(2):e1919706. doi:10.1001/jamanetworkopen.2019.19706

eTable. Absolute and Relative Mortality (SMR) Over Each Time Interval After Hip Fracture
eFigure. Age and Relative Mortality (Standardized Mortality Ratio) After Hip Fracture in A) Men and B) Women

This supplementary material has been provided by the authors to give readers additional information about their work.

© 2020 Yong E-L et al. *JAMA Network Open.*

eTable: Absolute and relative mortality (SMR) over each time interval after hip fracture.

	Mortality		SMR					
	MEN	WOMEN	MEN	95%CI		WOMEN	95%CI	
**3 m**	8.83	5.75	6.25	5.85	6.68	4.15	3.92	4.39
**6 m**	13.17	8.57	4.74	4.49	4.99	3.15	3.01	3.29
**9 m**	16.26	10.54	3.97	3.79	4.16	2.63	2.52	2.74
**1 y**	19.08	12.45	3.37	3.23	3.52	2.26	2.18	2.35
**2 y**	28.06	19.03	2.56	2.47	2.64	1.75	1.70	1.80
**3 y**	35.95	25.98	2.24	2.18	2.31	1.60	1.56	1.64
**4 y**	43.41	32.79	2.06	2.01	2.12	1.52	1.49	1.56
**5 y**	50.01	39.45	1.94	1.90	1.99	1.48	1.46	1.51
**6 y**	55.60	45.44	1.86	1.82	1.90	1.45	1.42	1.47
**7 y**	60.67	51.54	1.78	1.75	1.82	1.43	1.40	1.45
**8 y**	65.21	56.67	1.72	1.69	1.75	1.40	1.38	1.42
**9 y**	69.48	61.62	1.67	1.64	1.70	1.38	1.36	1.40
**10 y**	72.27	65.65	1.62	1.59	1.64	1.35	1.33	1.37
**11 y**	75.07	69.38	1.57	1.54	1.59	1.32	1.30	1.34
**12 y**	77.07	72.85	1.51	1.49	1.54	1.30	1.28	1.32
**13 y**	80.54	76.15	1.50	1.47	1.53	1.28	1.26	1.30
**14 y**	81.25	78.28	1.42	1.40	1.45	1.25	1.23	1.26

© 2020 Yong E-L et al. *JAMA Network Open.*

eFigure. Age and Relative Mortality (standardized mortality ratio) After Hip Fracture in A) Men and B) Women

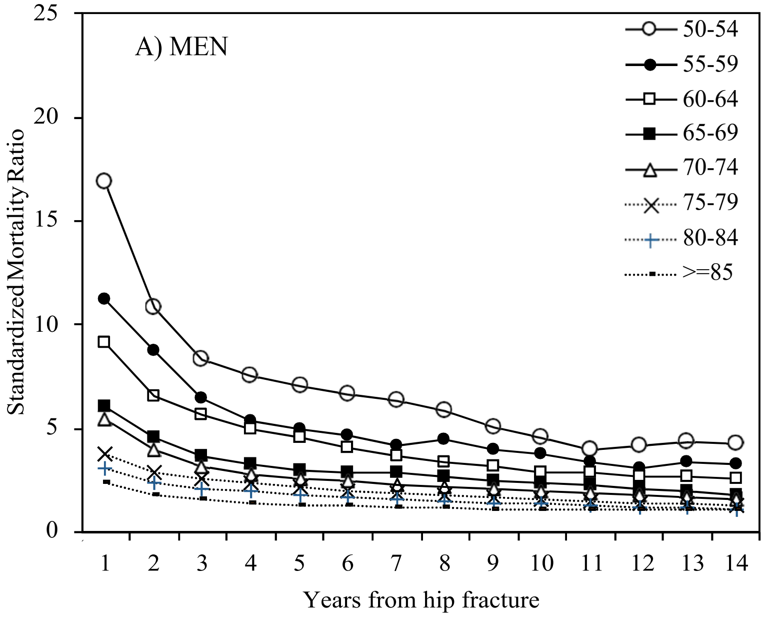

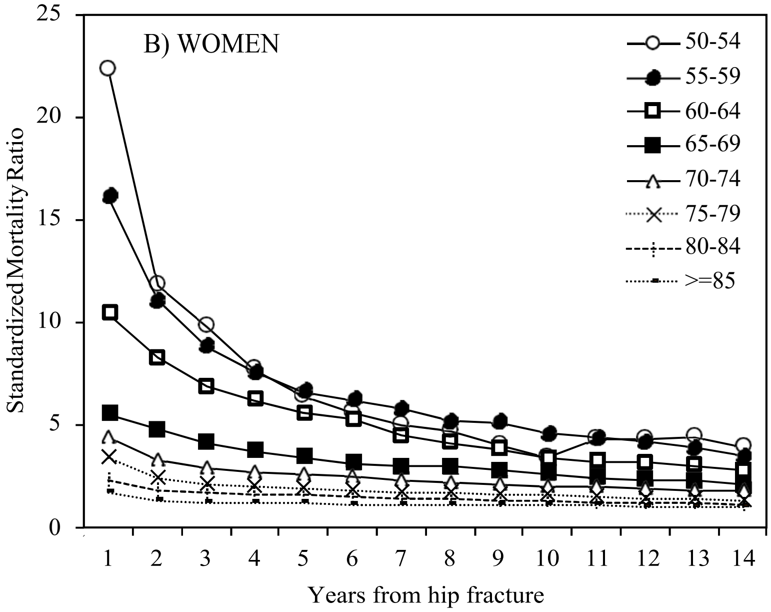

© 2020 Yong E-L et al. *JAMA Network Open.*
